# Dysfunction of lipid metabolism and lysosomal capacity: at the core of microglial aging?

**DOI:** 10.3389/fnagi.2026.1884230

**Published:** 2026-09-28

**Authors:** Eva Šimončičová, Marie-Ève Tremblay

**Affiliations:** 1 School of Medical Sciences, Faculty of Health, University of Victoria, Victoria, BC, Canada; 2 Center for Advanced Materials and Related Technologies (CAMTEC) and Institute of Aging and Lifelong Health (IALH), University of Victoria, Victoria, BC, Canada; 3 Department of Biochemistry and Molecular Biology, University of British Columbia, Vancouver, BC, Canada

**Keywords:** aging, microglia, microglial heterogeneity, lipid, lipid droplets, lysosome, synaptic plasticity

## Abstract

As primary immune cells of the central nervous system (CNS), microglia maintain immune surveillance, inflammation regulation, cell interactions and debris clearance. These dynamic and adaptive effector functions are reciprocally sustained by microglial metabolism. Lipids are emerging as a key programmer of microglial metabolic and functional flexibility in conditions of homeostasis, cellular stress, and pathology. The delicate equilibrium in microglial lipid homeostasis is dependent on the integrated activity of all cellular components, including the lysosome-lipophagy pathway. Continuous adaptations in microglial metabolism contribute to a cumulative imbalance between lysosomal network capacity and the efficacy of lipid turnover in aged microglia. The disequilibrium contributes to microglial fatigue and detrimental homeostatic activity associated with aging. Lipid droplet-accumulating microglia (LDAM) emerged as an age-associated example of these lysosome-lipid flux alterations. While characterized by dysregulated phagocytosis and pro-inflammatory intercellular signaling, the role of LDAM in the contexts of neuroplasticity, aging-related cognitive decline and frailty remains undetermined. This review discusses the immunometabolic profile of aging LDAM and their interactions with neurons and synapses, highlighting the impact of intercellular lipid signaling route.

## 1 Introduction

Microglia, the long-living immune effector cells of the central nervous system (CNS), have a pivotal role in maintenance of its homeostasis and adaptation (Elmore et al., 2018; Paolicelli et al., 2014; Sierra et al., 2014; Tremblay et al., 2010). Throughout life, these cells support maturation and survival of glial and non-glial cells, turnover of the myelin sheath and extracellular matrix, and phagocytose cellular debris, dying cells, and deleterious protein aggregates (Butovsky et al., 2006; Hagemeyer et al., 2017; Marquez-Ropero et al., 2025; Montilla et al., 2025; Nguyen et al., 2020; Sierra et al., 2014). A dynamic movement of their processes and complex network of surface pattern-recognition receptors enable microglia to continuously scan their parenchymal surroundings and rapidly mount effector functions (e.g., through migration, phagocytosis, trophic, cytokine and chemokine factor synthesis) to re-balance homeostasis (Davalos et al., 2005; Hanisch and Kettenmann, 2007; Nimmerjahn et al., 2005).

The metabolism of lipids is emerging as an essential regulator of microglial homeostatic activity and the ability to adapt their functional state to environmental changes across the lifespan (Choi and Mook-Jung, 2022; Loving et al., 2021; Prakash et al., 2025b; Traetta et al., 2025; Yang et al., 2022). Microglial lipids serve structural roles in membrane maintenance and remodeling and act as signaling mediators in key regulatory pathways, including energy production and inflammatory mediator release (Loving and Bruce, 2020; Pereira-Dutra et al., 2019; Prakash et al., 2025b; Traetta et al., 2025; Yang et al., 2022). Beyond their intrinsic lipid needs, microglia phagocytose lipid-rich substrates (i.e., myelin fragments, cell debris and synaptic elements) and generate intracellular lipid cargo through housekeeping autophagic processes (He et al., 2024; Lipinski et al., 2010; Safaiyan et al., 2016; Schafer et al., 2012; Xu et al., 2021; Zhou et al., 2023). Intracellular lipid droplets (LD), which store excess lipids, are emerging as essential regulators of microglial metabolic programming and functional capacity (Hsia et al., 2024; Lauro and Limatola, 2020; Prakash et al., 2025a; Shang et al., 2025; Tsap and Shcherbata, 2025). To maintain lipid balance and prevent lipotoxicity, microglia possess a complex feedback-regulated system for lipid uptake, processing, recycling, storage and export of excess lipids. Lysosomes, the cellular degradative and signaling hubs, lie at the center of these key lipid-handling pathways, both organizing lipid turnover and being modulated by the lipid status (Carmona-Gutierrez et al., 2016; He et al., 2024; Thelen and Zoncu, 2017). This bi-directional interplay underlying microglial metabolic programming is coordinated by a variety of lipid- and nutrient-sensing molecular players, notably involving mechanistic target of rapamycin complex 1 (mTORC1) or triggering receptor expressed on myeloid cells 2 (TREM2) (Abdulkhaliq et al., 2026; Feiten et al., 2026; Keane et al., 2021; Rabanal-Ruiz et al., 2017).

Aging drives maladaptive microglial functions contributing to inflammation and neurotoxicity in age-associated disorders (Antignano et al., 2023; Carr et al., 2025; Niraula et al., 2017). Dysfunctional lipid handling and lysosomal processing have been connected to this detrimental polarization, highlighting the relevance of this pathway for therapeutic targeting (Figure 1; Barrella et al., 2026; Carmona-Gutierrez et al., 2016; Cuervo and Macian, 2014; Guerrero-Navarro et al., 2022; Tan and Finkel, 2023; Tsap and Shcherbata, 2025). While significant advances have been made in the study of microglial lipid metabolism in contexts of aging-related diseases (Cantuti-Castelvetri et al., 2018; Montilla et al., 2025; Robb et al., 2025; Yang et al., 2014), our understanding of its changes over the course of physiological aging remains lacking, which creates a critical knowledge gap. Several single-cell analyses further indicate that microglial aging occurs in a CNS region-dependent manner (Allen et al., 2023; Grabert et al., 2016; Hahn et al., 2023; Tsai et al., 2025), raising fundamental questions: (1) is this regional heterogeneity associated with distinct microglial lipid signatures and/or lysosomal capacities; (2) do these putative baseline differences play a role in susceptibility to and extent of aging-related microglial dysfunction; and (3) is microglial lipid shift inherent and/or driven by environmental challenges? On a broader level, how these metabolic and functional aging microglial changes are translated into higher cognitive processes, and what role they play in their aging-associated decline and disease susceptibility, represents a crucial area for investigation.

**FIGURE 1:**
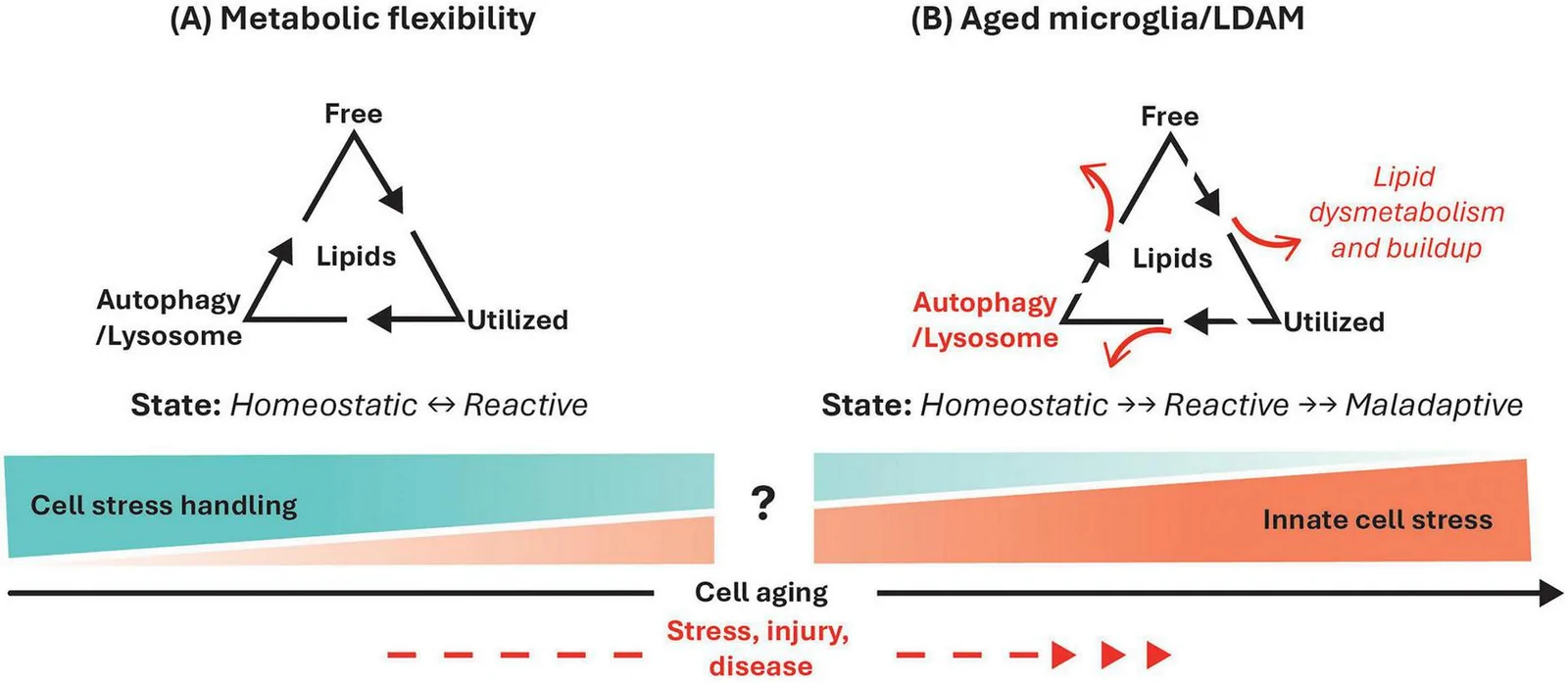
Microglial metabolic programming by lysosome-mediated lipid homeostasis and flux. **(A)** Lipid metabolism directs microglial fate and immune responsiveness by governing energy stores, membrane structure and signaling. As part of a coordinated regulatory network of lipid metabolism, autophagy-lysosome activity serves as a critical hub for lipid degradation and recycling. **(B)** The lysosomal function declines across the microglial lifespan. At an unknown threshold, the metabolic demand and intracellular stress overcome microglial ability to optimally support responses to homeostatic (e.g., lipoprotein turnover, cell waste and debris removal) or immune activators (e.g., hypoxia, cytokines, damage- and protein-associated molecular patterns, lipid and protein aggregates). Resulting dysregulation of intracellular lipid signaling pathways and accumulation of lipid burden drives maladaptive microglial function. Conditions requiring high and prolonged microglial metabolic activity, including chronic and/or severe stressors, injuries and diseases, promote acceleration of lipid dysregulation and cell aging.

The hippocampus is a core brain region for adult learning and memory formation, neurogenesis and mood regulation (Bettio et al., 2017; Šimončičová et al., 2024). Driven by aging-associated oxidative stress, neuroinflammation (defined here as inflammation taking place in the CNS) and reduced neuro/synaptic plasticity, the aging hippocampus undergoes extensive structural and functional alterations contributing to memory and cognitive decline (Bettio et al., 2017; Grabert et al., 2016). Adult microglia are indispensable for hippocampal synaptic transmission and experience-associated plasticity of neural circuits (e.g., through phagocytosis and release of cytokines or trophic mediators) (Sierra et al., 2014; Šimončičová et al., 2024; Tremblay, 2011; Wake et al., 2009). In the context of physiological aging, a significant portion of hippocampal microglia across species was reported to adopt a phenotype defined by notable lipid accumulation and lysosomal expansion (Haney et al., 2024; Marschallinger et al., 2020). Among the diverse spectrum of reported non-homeostatic microglial states to emerge with aging (Allen et al., 2023; Baldo et al., 2026; Bisht et al., 2016; Hammond et al., 2019; Rachmian et al., 2024; Safaiyan et al., 2021; Streit et al., 2004), these lipid droplet-accumulating microglia (LDAM) present an intriguing example of lipid dysmetabolism combined with functional deficiency (Haney et al., 2024; Marschallinger et al., 2020). Considerable attention is currently focused on understanding whether LDAM is a consequence and/or driver of processes associated with decline in hippocampal neuroplasticity and cognitive functioning. In this review, following a description of the microglial lipid-lysosome axis in homeostasis, we discuss microglial aging with a focus on LDAM and their putative contribution to age-related synaptic dysfunction.

## 2 Microglial lipid-lysosome axis in homeostasis

### 2.1 Lysosomal network and mTORC1 regulation

Two primary pathways for microglial delivery of lysosomal substrates include endo-/phagocytosis (extracellular components) and autophagy (intracellular components) (Figure 2; Guerrero-Navarro et al., 2022; Shin et al., 2021). Depending on the substrate type, microglial endocytosis utilizes clathrin-dependent or -independent pathways and non-selective macropinocytosis (Guerrero-Navarro et al., 2022; Shin et al., 2021). In comparison, the internalization of larger particles (e.g., cell debris, protein aggregates, myelin or synaptic fragments) during receptor-mediated phagocytosis requires more extensive remodeling of the microglial cytoplasmic membrane and cytoskeleton (Berglund et al., 2020; Davidson and Wood, 2020; Kessels et al., 2025). The resulting endosomes/phagosomes, respectively, bud off from the internal cytoplasmic membrane lamina to transfer the substrate for lysosomal digestion (Guerrero-Navarro et al., 2022; van Weering and Scheper, 2019). Enabling maintenance of intracellular homeostasis, the autophagic pathway delivers damaged organellar and membranous components, protein aggregates or LD (Lipinski et al., 2010; Moriwaki et al., 2024; Xu et al., 2021). In this process, an immature phagophore selectively forms around an intracellular cargo and gradually matures into a double-membraned delivery autophagosome. While distinct mechanisms, autophagy overlaps with endo-/phagocytosis on numerous levels, for instance, by using intracellular vesicle fusion/cargo exchange or recycling of phagocytic receptors (e.g., CD36, TREM2) (Carmona-Gutierrez et al., 2016; He et al., 2024; Lucin et al., 2013; van Weering and Scheper, 2019). For substrate degradation, vesicles of both pathways fuse with lysosomal organelles in a process which involves cargo sorting and lysosomal membrane alteration (Bright et al., 2016; van Weering and Scheper, 2019). The free lysosomal pool is maintained via biogenesis or a process of reformation, where an empty protolysosome buds off from a mature lysosome (He et al., 2024). The low acidity of the lysosomal lumen (pH 4.5–5.5) regulates the catabolic activity of a spectrum of hydrolytic enzymes and their co-activators. The luminal pH is maintained by membrane-associated ion channels and ATP-dependent proton pumps [e.g., vacuolar-type ATPase (V-ATPase)] (Colacurcio and Nixon, 2016; Moriwaki et al., 2024; Wang B. et al., 2024). The products of hydrolytic macromolecule breakdown are exported to the cytoplasm or to downstream organelles via membrane transporters and reutilized for biosynthesis, energy production, renewal, signaling or storage.

**FIGURE 2:**
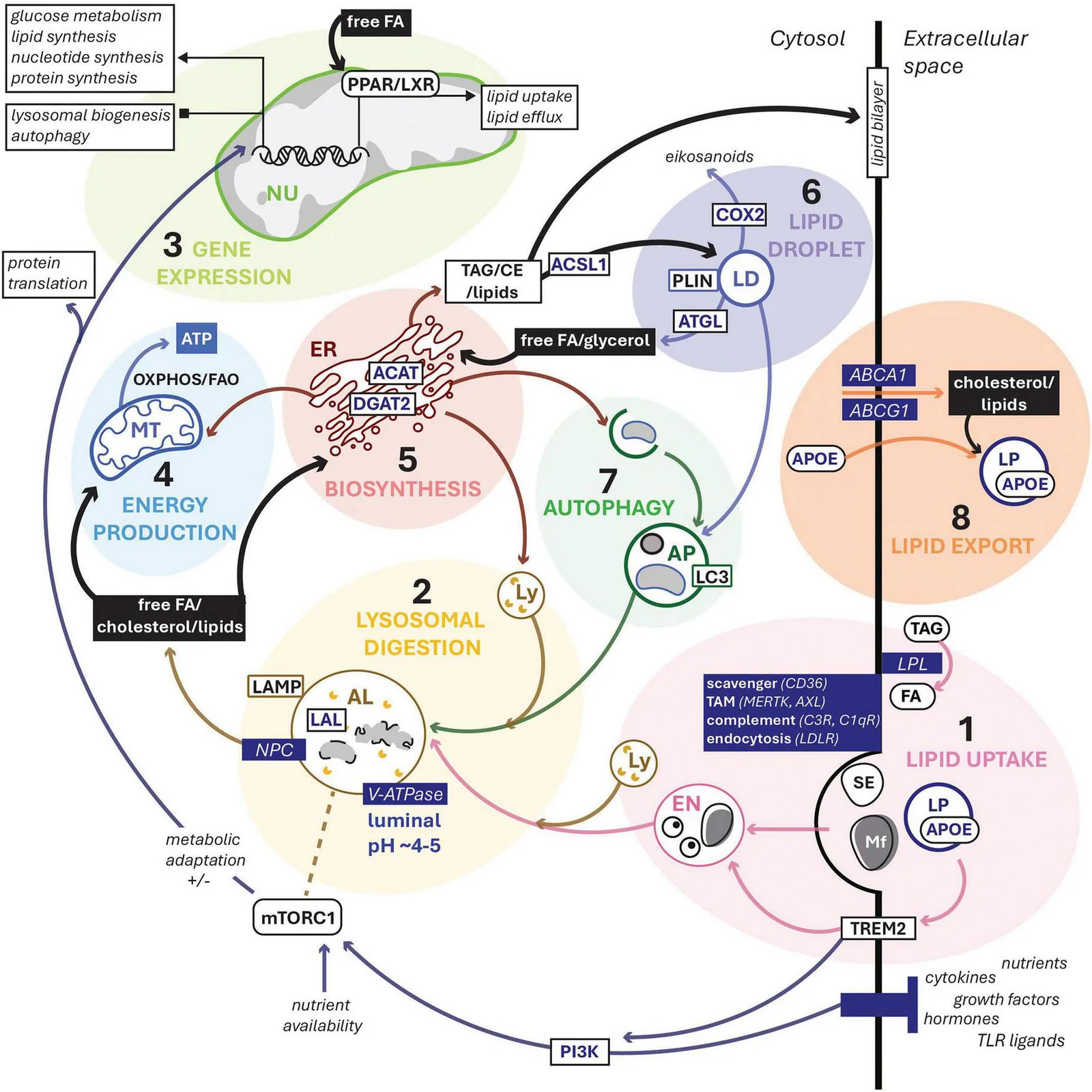
Lysosome-mediated lipid handling regulates microglial metabolism and function. Lipids are maintained in a constant turnover orchestrated across all cellular compartments. (1) A wide range of surface receptors mediates uptake of lipids or various lipid-associated substrates (e.g., scavenger, TAM-family, complement, endocytic) or their regulation (e.g., TREM2). LPL is a surface-associated enzyme that supports lipid uptake through hydrolysis of TAG and tethering of lipid substrates and uptake receptors. (2) Lysosomes fuse with phago-/endo-/autophagosomes for enzymatic hydrolysis of complex and bound lipids in their lumen (e.g., TAG, CE), providing a source of free fatty acids and cholesterol for other cellular pathways. (3) The PI3K-mTORC1 pathway integrates signals from a spectrum of surface receptors for cytokines, nutrients, growth factors, hormones, immune ligands, phagocytosis-associated receptors (e.g., TREM2), with the nutritional state of the cell. If activated, mTORC1 enhances anabolic processes and dynamically regulates activation of catabolic processes (e.g., autophagy and lysosomal activity) to promote microglial growth, survival and function. PPAR/LXR are nuclear transcription factors reacting to fluctuations in the cytosolic lipid pool by upregulation of lipid flux-associated genes. Cytosolic free FA/cholesterol/other lipid metabolites are utilized for, e.g., (4) mitochondrial FAO whose metabolites support respiration and ATP production or (5) ER-mediated enzymatic synthesis of complex lipids. These complex lipids (e.g., TAG, CE, sphingolipids, or phospholipids) are utilized for the function and structure of organelles as well as the cytoplasmic membrane. (6) To prevent lipotoxicity, excess neutral lipids can be stored in ER-derived LDs. To fuel the metabolic processes during immune response or cellular stress, LDs can be broken down through cytosolic lipolysis (mediated via ATGL) or (7) via selective autophagy (lipophagy) and lysosomal digestion. (8) Cholesterol efflux is mediated via membrane-bound transporters (ABCA1/ABCG1), in a process where APOE molecules serve as extracellular lipid carriers. Cell-associated structures: AL, autolysosome; AP, autophagosome; EN, endo-/phagosome; ER, endoplasmic reticulum; LD, lipid droplet; LP, lipoprotein; Ly, lysosome; Mf, myelin fragment; MT, mitochondria; NU, nucleus; SE, synaptic element. Enzymes: ACAT, acyl-CoA:cholesterol acyltransferase; ACSL1, acyl-CoA synthetase long chain family member 1; ATGL, adipose triglyceride lipase; DGAT2, diacylglycerol o-acyltransferase 2; LAL, lysosomal acid lipase; LPL, lipoprotein lipase. Membrane-bound proteins: LAMP, lysosome-associated membrane protein; LC3, microtubule-associated protein 1a/1b-light chain 3; PLIN, perilipin. Molecules and metabolic processes: APOE, apolipoprotein E; ATP, adenosine triphosphate; CE, cholesterol ester; FA, fatty acid; FAO, fatty acid oxidation; OXPHOS, oxidative phosphorylation; PI3K, phosphatidylinositol 3-kinase; TAG, triglycerides. Receptors and transporters: ABCA1, ATP-binding cassette transporter A1; ABCG1, ATP-binding cassette sub-family G member 1; CD36, cluster of differentiation 36 (scavenger receptor); LDLR, low-density lipoprotein receptor; LXR, liver X receptor; mTORC1, mechanistic target of rapamycin complex 1; NPC, Niemann-Pick C1/2 cholesterol transporters; PPAR, peroxisome proliferator-activated receptor; TAM, TAM-family receptors (MERTK, AXL); TLR, Toll-like receptor family; TREM2, triggering receptor expressed on myeloid cells 2; V-ATPase, vacuolar-type ATPase.

The integrity and stability of the lysosomal membrane is maintained by an abundance of proteins, including lysosome-associated membrane proteins 1 and 2 (LAMP1 and -2) (Huynh et al., 2007; Schneede et al., 2011). Absence of these membrane proteins triggered autophagy flux impairment and endo/lysosomal cholesterol buildup in murine fibroblasts *in vitro* (Eskelinen et al., 2004; Reynolds et al., 2024). Beyond the digestive function in the lumen, the lysosomal membrane serves as a signaling platform integrating signals about nutrient availability, autophagic recycling, cellular growth and metabolism. This function is largely mediated by mTORC1, a master protein kinase complex (Ben-Sahra and Manning, 2017; Byles et al., 2013; Rabanal-Ruiz et al., 2017). As a nutrient sensor and metabolic regulator, mTORC1 is generally inhibited during starvation or cellular stress, and activated in nutrient-rich conditions by binding to the lysosomal membrane (Ben-Sahra and Manning, 2017; Byles et al., 2013). Negatively regulated by mTORC1, activation of transcription factor EB (TFEB) triggers the transcription of genes associated with lysosomal biogenesis and function (Bernier et al., 2020b; Papadopoli et al., 2019; Rabanal-Ruiz et al., 2017). TFEB is also a key positive regulator of autophagy-associated genes through activation of the Unc51-like autophagy activating kinase 1 (ULK) complex (Lin et al., 2023). When activated, mTORC1 inhibits catabolic processes and facilitates gene expression associated with protein, nucleotide and lipid synthesis as well as glycolysis (Ben-Sahra and Manning, 2017).

Given the nature of their function, lysosomes are continuously exposed to potential damage to their structure, which may trigger negative outcomes on their metabolic sensing, digestion, recycling, or signaling. As such, a tight control of lysosomal integrity through several levels of intertwined quality control mechanisms, including biogenesis, repair, or turnover, is essential for their function [as comprehensively reviewed by He et al. (2024)]. This is specifically relevant for the maintenance of lipid homeostasis in microglia, whose extensive uptake of lipids and lipid-rich substrates may place substantial demands on lysosomal activity.

### 2.2 Lipid uptake and export

The mammalian brain contains a high abundance and diversity of lipid species (∼700 species in mouse), second only to adipose tissue (Fitzner et al., 2020). Cholesterol, phospholipids, sphingolipids, fatty acids (FA) and neutral lipids [triglycerides (TAG) or cholesteryl esters (CE)] constitute primary brain lipids (Loving and Bruce, 2020; Simons and Ehehalt, 2002; Tsap and Shcherbata, 2025). In homeostasis, major sources of microglial extracellular lipid exposure include (1) myelin, enriched in cholesterol and phospholipids; (2) apoptotic cells and synaptic elements; or (3) lipoprotein particles (Loving and Bruce, 2020; Simons and Ehehalt, 2002; Tsap and Shcherbata, 2025). Microglia utilize a variety of endocytic-, phagocytic- and pattern recognition receptor (PRRs)-mediated pathways to regulate the clearance of lipids and lipid-enriched substrates from the extracellular space (Gabandé-Rodríguez et al., 2020; Guerrero-Navarro et al., 2022).

TREM2 is a transmembrane receptor, which is primarily expressed by microglia in the CNS (Feiten et al., 2026; Nugent et al., 2020). Through recognition of a spectrum of ligands including polyanions, lipids and lipoproteins, apoptotic cell-related phosphatidylserine, bacterial or amyloid beta (Aβ) molecules, TREM2 serves as a major lipid- and damage-sensing receptor (Feiten et al., 2026; Kober and Brett, 2017). Its activation promotes adaptor protein DAP12 (TYROBP) phosphorylation and initiation of downstream signaling pathways supporting phagocytosis, metabolism, cell survival and regulation of inflammatory activity (Feiten et al., 2026; Kober and Brett, 2017). In terms of lipid-lysosomal biology, TREM2 acts as a positive upstream regulator of mTORC1, thus coupling lipid sensing with metabolic reprogramming (Feiten et al., 2026; Kober and Brett, 2017; Qiao et al., 2024). TREM2 is also considered a driving element for microglial transformation into disease-associated microglia (DAM), a pro-phagocytic microglial response to damage-associated molecular patterns, protein aggregates and altered lipid homeostasis in aging and disease (Keren-Shaul et al., 2017; Krasemann et al., 2017; Poliani et al., 2015). Moreover, TREM2 regulates microglial gene expression of Apolipoprotein E (APOE), an extracellular lipid carrier molecule (Nugent et al., 2020; Shi and Holtzman, 2018; Victor et al., 2022). APOE, whose genetic variants influence Alzheimer’s disease (AD) risk, binds to low-density lipoproteins (LDL) or cholesterol-rich particles and enables their receptor-mediated uptake (de Leeuw et al., 2022; Haney et al., 2024; Shi and Holtzman, 2018).

In concert with TREM2, the TAM family receptors (MerTK and Axl) or cluster of differentiation 36 (CD36) participate in phagocytosis of lipids/lipid-rich particles and microglial metabolic programming in a context-dependent manner (Coraci et al., 2002; Fourgeaud et al., 2016; Healy et al., 2016). Lipoprotein internalization is also mediated through endocytic receptor pathways, which include low-density lipoprotein receptor (LDLR), very low-density lipoprotein receptor (VLDLR) or low-density lipoprotein receptor-related protein 1 (LRP1) (Brifault et al., 2017; Pocivavsek et al., 2009). With its expression elevated in microglia responding to disease (e.g., AD pathology), the role of extracellular lipoprotein lipase (LPL) has been increasingly discussed in the context of microglial lipid programming (Cheng and Ho, 2025; Loving et al., 2021). Beyond its canonical function of mediating extracellular hydrolysis of TAG-enriched particles into free FA, LPL was proposed to facilitate interactions between extracellular lipids and cell surface receptors (Kumari et al., 2021; Loving et al., 2021). Its knockout (KO) in primary mouse microglial cells was associated with impaired microglial lipid sensing and turnover, lipid accumulation, and synthesis of lipid-derived inflammatory mediators (Loving et al., 2021). This indicates both a critical role for LPL in lipid flux, as well as a significant dependence of microglial fitness on an unperturbed balance between individual processes of lipid metabolism. To counter lipid intake and intracellular lipid processing, microglia utilize a family of ATP-binding cassette transporters (ABC), such as ABCA1 and ABCG1, for excess cholesterol/lipid efflux (Karasinska et al., 2013; Li et al., 2024b; Mauerer et al., 2009). The role of ABC transporters lies primarily in the transfer of cholesterol/lipids on extracellular high-density APOE-lipoprotein particles (Hirsch-Reinshagen et al., 2004). As an intracellular regulatory system, transcription factors liver X receptor (LXR) and peroxisome proliferator-activated receptor (PPAR) sense lipid fluctuations and control microglial expression of various lipid metabolism-associated genes, including ABC transporters (Hong and Tontonoz, 2008). Associated with the promotion of anti-inflammatory microglial activity (Bilotta et al., 2020; Cantuti-Castelvetri et al., 2018; Pearson et al., 2024), this regulatory mechanism further strengthens the connection between microglial metabolic capacity and function.

### 2.3 Lysosome-mediated lipid catabolism and recycling for cellular mechanisms

Microglial lysosomes contain a diverse range of lipid-degrading enzymes (Quick et al., 2023). A rate-limiting esterase in cholesterol trafficking is lysosomal acid lipase (LAL), which converts imported CE and TAG into free cholesterol, FA and glycerol (Barnett et al., 2025; Hu et al., 2026). Sphingomyelin, a structural component of the myelin sheath, is hydrolyzed by acid sphingomyelinase, generating ceramides and phosphocholine (Allende et al., 2021; Guerrero-Navarro et al., 2022). Acid ceramidase further catabolizes ceramides into FA and sphingosine (Allende et al., 2021; Guerrero-Navarro et al., 2022). Lipid cell profiling revealed that isolated adult murine brain microglial cells are particularly enriched in sphingolipid metabolism compared to other cell types (Fitzner et al., 2020). As sphingolipids are an essential component of the plasma membrane, this is likely a combined outcome of microglial extensive and dynamic arborization, and phagocytosis-mediated clearance of membrane structures like myelin and cell debris. Other enzymes include those involved in the catabolism of glycosphingolipids or phospholipases, such as beta-glucocerebrosidase (GCase), galactocerebrosidase, or beta-galactosidase (Guerrero-Navarro et al., 2022; Quick et al., 2023; Zhou et al., 2019). Lysosomal export of cholesterol and other lipids (e.g., sphingosine, gangliosides, sphingomyelin) relies on a coordinated activity of Niemann-Pick type C cholesterol transporters 1 and 2 (NPC1 and NPC2) (Colombo et al., 2021; Gabandé-Rodríguez et al., 2020; Zareba et al., 2024). Apart from transporters, membrane proteins are also involved in the regulation of enzymatic activity. Through support of GCase or via stabilization of luminal phospholipid bis(monoacylglycero)phosphate (BMP), progranulin (PGRN) is involved in lysosomal processing of membrane-bound ganglioside and cholesterol trafficking (Showalter et al., 2020; Zhou et al., 2019).

Lysosomes also form membrane contact sites with other organelles, including the endoplasmic reticulum (ER), Golgi apparatus, mitochondria, peroxisomes, and LDs (Bandyopadhyay et al., 2024). The proximity and structure of these inter-organellar contacts enable bidirectional exchange of lipids and ions between compartments, which facilitates lipid flux, homeostasis, and communication (Bandyopadhyay et al., 2024). This places the lysosomal axis within an integrated organellar network that cohesively organizes lipid homeostasis and metabolic adaptation on a cellular level. Briefly, the microglial ER is of particular significance as an organelle involved in the biogenesis of lysosomes and synthesis of lysosomal enzymes (Bajaj et al., 2019; Guerrero-Navarro et al., 2022; Wilfling et al., 2014). Moreover, it also represents the primary platform for autophagic phagophore budding and utilizes free lipids for *de novo* synthesis of phospholipids, cholesterol, and sphingolipid precursors, or re-packaging into neutral lipids and LD formation (Yang et al., 2022; Traetta et al., 2025; Tsap and Shcherbata, 2025). These processes are catalyzed by the activity of key regulatory enzymes such as diacylglycerol acyltransferase-2 (DGAT2), acyl-CoA:cholesterol acyltransferase (ACAT) and acyl-CoA synthetase long-chain family member 1 (ACSL1) (Al-Rashed et al., 2023; Prakash et al., 2025a; Sung et al., 2025).

### 2.4 Lipid droplets and lipophagy

Generated by budding off from the ER membrane, LD store neutral lipids (TAG, CE) within a hydrophobic phospholipid monolayer (Wilfling et al., 2014). The LD surface is decorated by a range of specific proteins, including a family of perilipins (PLIN). LD stability is regulated by the ratio of their luminal lipid classes and activity of PLIN proteins, which also enable LD signaling and tethering to other organelles, i.e., mitochondria (Huang et al., 2026). Lysosome-mediated turnover of LDs is mediated through lipophagy, a selective form of autophagy, which is under the control of the mTORC1-TFEB pathway (Schott et al., 2022; Shang et al., 2025). Another path for LD lipolysis is mediated by the activity of cytosolic adipose triglyceride lipase (ATGL) (Chandak et al., 2010; Robb et al., 2025). Its KO in primary murine macrophages showed an impaired intracellular lipid flux and deposits (Chandak et al., 2010). ATGL regulated acute pro-inflammatory responses of neonatal murine microglial cells to lipopolysaccharide (LPS) *in vitro*. Its microglia-specific inhibition attenuated LPS-induced sickness and pro-inflammatory signaling in adult male mice (Robb et al., 2025). This effect was suggested to occur via loss of FA for biosynthesis of pro-inflammatory mediators such as eicosanoids and ceramides (Robb et al., 2025). Further revealing its broad impact on management of cellular lipid catabolism and energy stores, mTORC1 activation was linked with ATGL inhibition in adipocytes (Chakrabarti et al., 2010) and a negative regulation of PPARγ signaling in hepatocytes of mice (Sengupta et al., 2010).

Originally considered storage vesicles, LD have since emerged as dynamic lipid-rich organelles involved in the regulation of lipid homeostasis, energy production and signaling across cell types (Wilfling et al., 2014). Macrophages were also shown to induce lipid reprogramming and LD turnover as part of an evolutionarily conserved immune response to bacterial or viral stimuli [e.g., mediated by Toll-like receptor 4 (TLR4)] or when facing high levels of lipid-rich debris (e.g., atherosclerosis) (Hsia et al., 2024; Pereira-Dutra et al., 2019; Robb et al., 2025; Shang et al., 2025; Stephenson et al., 2025; Wang et al., 2023). This pathway is expected to serve as a protection from lipotoxicity as well as an energetic source for mitochondria and functional transformation associated with acute immune response (Hsia et al., 2024; Sung et al., 2025; Tsap and Shcherbata, 2025).

### 2.5 Roles of lipids in microglial homeostasis and function

The extensive array of free lipid species or lipid-derived molecules is essential for microglial structure, signaling and metabolism. On a structural level, phospholipids, glycolipids, and sterols constitute fundamental components of microglial cellular membranes. Their ratio regulates membrane permeability, fluidity, curvature and stability (Loving and Bruce, 2020; Prakash et al., 2025b; Simons and Ehehalt, 2002; Tsap and Shcherbata, 2025). Particularly enriched in cholesterol and sphingolipids are lipid rafts, which form a scaffold for clustering of receptors and signaling proteins. These membrane microdomains then serve as hubs for vesicle trafficking and cytoskeletal communication, and as organizing sites for glial inflammatory responses (Miller et al., 2020; Simons and Ehehalt, 2002). The role of membrane lipids extends beyond structural scaffolding, as these components can be converted into potent signaling messengers that regulate microglial behavior [e.g., phosphatidylinositol 4,5-bisphosphate (PIP2)]. Phosphoinositide 3-kinase (PI3K) is a lipid kinase which cleaves PIP2 into phosphatidylinositol 3,4,5-trisphosphate (PIP3), an activator of protein kinase B (AKT) (Cianciulli et al., 2020). PI3K/AKT pathway is a downstream signaling axis for a spectrum of microglial surface receptors, including TREM2, growth factor receptors, cytokine receptors, and TLRs (Cianciulli et al., 2020; Han et al., 2022; Haney et al., 2024). Acting on mTORC1, it is essential for dynamic adaptation of microglial metabolism and function in response to extracellular conditions (Cianciulli et al., 2020; Song et al., 2025). TREM2 and TLRs also signal on transmembrane protein phospholipase C gamma 2 (PLCγ2), which is dominantly expressed in microglia in the CNS and competes for PIP2 substrate (Bedford et al., 2025; Li et al., 2022). Its lipid substrates, diglyceride (DAG) and inositol 1,4,5-triphosphate (IP3), impact ER-related calcium release, thus regulating cell survival and phagocytosis (Li et al., 2022). Beyond these functions, lipids concurrently serve as substrates for cellular bioenergetics. Mitochondrial ACSL1 converts free FA into Acyl-CoA, which becomes further metabolized into Acetyl-CoA within the mitochondrial matrix (FA oxidation; FAO). Resulting Acetyl-CoA aids the activity of tricarboxylic/citric acid (TCA) cycle as substrate for the generation of metabolites for oxidative phosphorylation (OXPHOS) and adenosine triphosphate (ATP) production (Al-Rashed et al., 2023; Martínez-Reyes and Chandel, 2020).

In this manner, lipids participate in the regulation of key processes underlying microglial effector functions; an effect dependent on lipid type availability and exposure. For instance, systemic abundance of saturated FA (SFA) in mice on a high-fat diet regimen triggered microglial adoption of a pro-inflammatory profile (Valdearcos et al., 2014). SFA mode of action was connected to oxidative stress, nuclear factor kappa B (NF-κB), and the NOD-like receptor pyrin domain-containing 3 (NLRP3) inflammasome in primary mouse microglia and macrophages (Valdearcos et al., 2014; Wen et al., 2011). In a model of demyelination, sustained uptake of myelin was linked to upregulation of stearoyl-CoA desaturase 1 (SCD1) in isolated mouse microglial cells and bone marrow-derived macrophages (BMDM) (Bogie et al., 2020). Catalyzing SFA conversion to mono-unsaturated FA (MUFA) in the ER, elevated SCD1 decreased cholesterol flux, and induced a shift to a lesion-promoting microglial phenotype (Bogie et al., 2020). On the contrary, polyunsaturated FA (PUFA) are essential components of cell membrane lipids (Harayama and Shimizu, 2020). Critically, n-3 PUFA are sensitive to reactive oxygen species (ROS)-mediated lipid peroxidation (Sies et al., 2022), indicating vulnerability of microglial membrane integrity and functional dynamics to oxidative stress (Loo et al., 2025; Loving and Bruce, 2020; Mortensen et al., 2023). Agonism of n-3 PUFA (e.g., docosahexaenoic acid) receptor attenuated NF-κB-associated pro-inflammatory responses in primary mouse microglial cells and mouse sickness response upon LPS exposure *in vivo* (Nakajima et al., 2023; Tremblay et al., 2016). Conversely, low maternal levels n-3 PUFA were reported to influence the offspring’s microglial lipid profile in mice, driving aberrant synaptic phagocytosis in the developing hippocampus (Madore et al., 2020). By contrast, n-6 PUFA, such as arachidonic acid, are key precursors for the synthesis of pro-inflammatory eicosanoids [(e.g., prostaglandin E2 (PGE2)] (Minhas et al., 2021).

### 2.6 Conceptual regional heterogeneity in microglial lipid handling

Given its role in sustaining dynamic microglial adaptability to diverse conditions, lipid handling likely does not occur uniformly across the heterogeneous microglial population. While direct evidence linking variable brain regions to distinct microglial lipid signatures remains limited, several observations indicate the biological plausibility of varying lipid metabolism among the microglial population. Quantitative shotgun lipidomics revealed distinct brain region-dependent lipid profiles in homogenized brain samples of adult wild-type mice (Fitzner et al., 2020), non-human primates and healthy adult humans (Osetrova et al., 2024; Söderberg et al., 1990). Highlighting structural differences between the white and gray matter, these studies indicated the presence of myelin as a dominant driver underlying regional lipidome differences (Fitzner et al., 2020; Osetrova et al., 2024). The human study further associated lipidome signatures with hierarchical information processing and functional connectivity, suggesting that regional lipid composition reflects functional (and structural) specializations of the environment (Osetrova et al., 2024). Lipids positively correlating with this hierarchical categorization were enriched in sphingomyelins and, among other glial cells, assigned to microglia (Osetrova et al., 2024). The general lipid signature of primary mouse microglial cells was marked by high concentrations of sphingomyelin and phosphatidylglycerol (Fitzner et al., 2020). Further proteomic evidence in the same model indicated enrichment in lipid degradation, prostaglandin, arachidonic, and sphingolipid metabolism pathways (Fitzner et al., 2020); however, regional microglial differences were not measured. Adult mouse microglia also displayed region-dependent transcriptomic signatures, which were driven by differences in immunoregulatory and bioenergetic pathways (Allen et al., 2023; Grabert et al., 2016; Hart et al., 2012). Spatial proteomics profiling additionally revealed a continuum of functional microglial immune states shaped by local cues at regional and sub-regional levels in post-mortem brain tissue from cognitively healthy aged individuals (Böttcher et al., 2020; Mrdjen et al., 2025).

With aging, several transcriptomic studies reported spatial differences in the magnitude and rate of microglial gene expression in mouse (Allen et al., 2023; Grabert et al., 2016; Hahn et al., 2023). Commonly deregulated gene pathways were linked with antigen presentation, interferon response, complement cascade and microglial activity (e.g., *Trem2*). Particularly, murine white matter-associated regions were consistently identified as more susceptible to aging and presenting with more profound aging-associated alterations in microglial signature relative to the gray matter (Allen et al., 2023; Hahn et al., 2023). This aligns with the observation of white and gray matter-specific patterns of changes in the lipidome of aged mice (Fitzner et al., 2020). There is also some early evidence for sex differences in aging-related regional gene expression, particularly in the hypothalamus. Generally, male mice upregulated genes involved in lipid metabolism and stress response, while female mouse genes were more strongly enriched in immune and inflammatory pathways (Hahn et al., 2023). These findings align with the observation of distinct transcriptional and epigenetic aging trajectories between sexes (Li et al., 2023). However, research is warranted to explore and characterize these possible sex differences, particularly as many -omics studies pool sexes together in their analysis.

In conclusion, the putative existence of innate heterogeneity in lipid handling programs could partially explain the variability in microglial functions across physiological and disease contexts; however, additional contributions from the microglial environment cannot be excluded. Advanced understanding of these relationships represents an important step for the definition of microglial function and the discovery of microglia-based interventions for cognitive aging and age-related pathological conditions.

## 3 LDAM and aging-associated patterns in microglial lipid-lysosome axis

### 3.1 Lysosomal dysfunction and accumulation of lipids

Across tissues, aging imposes a sustained burden on cellular maintenance systems, resulting in cellular stress and a gradual loss of metabolic balance and proteostasis (Costa et al., 2021; Goyal et al., 2017; Niraula et al., 2017; Peters, 2006). In microglia, breakdown of the lipid-lysosomal axis integrity is characterized by an impaired autophagic flux and lysosomal dysfunction, which reshapes their metabolic identity (Figure 3; Burns et al., 2020; Carr et al., 2025; Marschallinger et al., 2020; Safaiyan et al., 2016; Tremblay et al., 2016). The size of microglial LAMP1^+^ or CD68^+^ lysosomes markedly expands, and their cargo becomes progressively more differentiated with age, as shown in aged mouse and human brain samples using electron microscopy *in situ* (Burns et al., 2020; Marschallinger et al., 2020). Accompanying this expansion is the buildup of lipofuscin (LF), an insoluble and dense aggregate of cross-linked and oxidized lipids, proteins, carbohydrates and metals (Burns et al., 2020; Safaiyan et al., 2016; Stillman et al., 2023). Up to 70%–80% of microglia isolated from whole brains of wild-type mice and non-human primates were found to display an increasing rate of LF-linked autofluorescence between adult and aged stages of life (Burns et al., 2020). A higher load and earlier onset of LF accumulation was also reported in murine white compared to gray matter *in situ* (Burns et al., 2020; Safaiyan et al., 2016; Stillman et al., 2023). These observations indicate that (1) alterations in microglial lysosomal capacity may have an early onset and (2) a diverse capacity for LF accumulation exists among the microglial population. In the latter case, whether that is a feature of microglial state functional specialization, innate lysosomal capacity and/or interactions with the environment needs to be disentangled.

**FIGURE 3:**
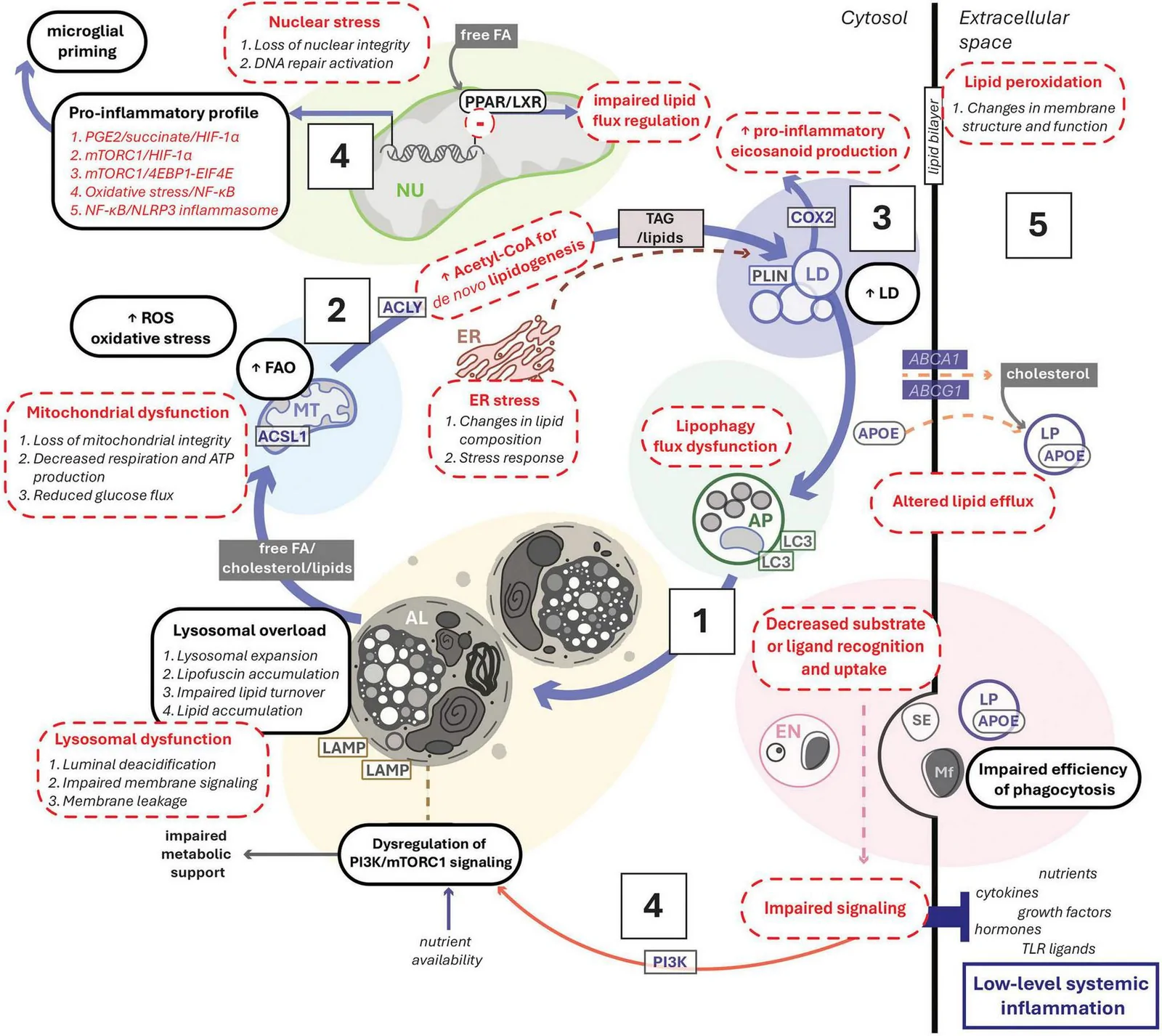
Conceptual framework of reported (black/black rectangles) and indicated (red/red dashed rectangles) molecular processes modulating priming and functional profile of age-associated LDAM. Aging promotes dysregulation of multiple networks involved in the regulation of lipid flux; however, the involved initial and sequential molecular mechanisms are unclear. (1) LDAM show LD accumulation and increased lysosome-mediated lipophagic turnover. As lysosomal function declines with aging and digestion residue buildup, the enhanced lipid flux contributes to lipid accumulation, tentatively exacerbating lysosomal aging dysfunction. (2) Aging microglia show a decline in mitochondrial integrity, decreased respiration and a metabolic shift from OXPHOS to glycolysis. LDAM were connected to elevated FAO and ACLY-mediated de novo lipogenesis, indicating upregulation in lipolytic pathways to support the cell’s function and survival. These contribute to oxidative stress via production of reactive oxidative and nitric substrates in LDAM. Of note, the ER is a hub for lipid synthesis and LD formation; however, its role in this process and linkage to its putative dysfunction in aging LDAM is undetermined (dashed arrow). (3) An elevated number of LD may be contributing to COX2-mediated synthesis of pro-inflammatory eicosanoids. (4) Microglial aging was also linked to an upregulation of the PI3K/mTORC1 metabolic sensor pathway, as a possible outcome of continuous exposure to innate immune activators, systemic inflammaging, as well as lipid overload. Eicosanoid- or mTORC1-induced cumulative activation of pro-inflammatory cytokine transcription via HIF-1α represent some of the putative pathways mediating the converging inflammatory profile and priming of LDAM. mTORC1 activity was also associated with cytokine protein translation via suppression of 4E-BP1 and activation of translation initiation factor eIF4E in aged microglia. Lastly, elevated oxidative stress induces translocation of NF-kB to the nucleus and transcription of pro-inflammatory cytokines and chemokines. Alternatively, oxidative stress and lysosomal integrity impairment may drive activation of NLRP3 inflammasome assembly and caspase-dependent IL-1β signaling. Another putative pathway also includes reduced PPAR/LXR-mediated transcription of genes involved in lipid flux. (5) Oxidative stress and impaired lipid flux may contribute to elevated peroxidation of lipids in cellular membranes, including the cytoplasmic membrane. These changes may affect its fluidity and structure, e.g., in lipid rafts, thus impacting receptor function, signaling and membrane-mediated processes, such as cellular movement or phagocytosis. LDAM were thus associated with delayed and decreased capacity for phagocytic cargo uptake. Considering the dependence of optimal lipid flux on optimal activity of all its components, it is likely that cholesterol efflux is altered in LDAM. Cell-associated structures: AL, autolysosome; AP, autophagosome; EN, endo-/phagosome; ER, endoplasmic reticulum; LD, lipid droplet; LP, lipoprotein; Ly, lysosome; Mf, myelin fragment; MT, mitochondria; NU, nucleus; SE, synaptic element. Enzymes: ACLY, ATP citrate lyase; ACSL1, acyl-CoA synthetase long chain family member 1; COX2, cyclooxygenase-2; LPL, lipoprotein lipase. Membrane-bound proteins: LAMP, lysosome-associated membrane protein; LC3, microtubule-associated protein 1a/1b-light chain 3; PLIN, perilipin. Molecules and metabolic processes: APOE, apolipoprotein E; ATP, adenosine triphosphate; CE, cholesterol ester; FA, fatty acid; FAO, fatty acid oxidation; HIF-1α, hypoxia-activated factor 1 alpha; OXPHOS, oxidative phosphorylation; PI3K, phosphatidylinositol 3-kinase; TAG, triacylglycerides; ROS, reactive oxidative species. Receptors and transporters: ABCA1, ATP-binding cassette transporter A1; ABCG1, ATP-binding cassette sub-family G member 1; LAMP, lysosome-associated membrane protein; LXR, liver X receptor; mTORC1, mechanistic target of rapamycin complex 1; PPAR, peroxisome proliferator-activated receptor; TLR, Toll-like receptor family.

Human and murine LDAM are further characterized by large lipid bodies occupying the lysosomal compartments alongside lipofuscin (Marschallinger et al., 2020); however, the mechanistic driver of their accumulation is unclear. LF and diverse storage material, including yet undigested membranous structures, lipid and protein aggregates identified *in situ*, may contribute to decline in lysosomal function and exacerbate toxic substrate storage (Burns et al., 2020; Guerrero-Navarro et al., 2022; Moreno-García et al., 2018; Safaiyan et al., 2016). As a source of redox-active metals, LF impaired membrane integrity and attenuated activity of lysosomal proton pumps in primary human retinal pigment epithelial cells (Bergmann et al., 2004; Colacurcio and Nixon, 2016). The progressive alkalinization of the lysosomal lumen may interfere with the activity of pH-dependent lipases and lipid transporters in microglia with aging (Barrella et al., 2026; Quick et al., 2023). Mimicking neurological symptoms of aging and characterized by toxic lipid buildup are lysosomal storage disorders, a family of rare hereditary diseases with genetic defects in lysosome-related enzymes and proteins (Guerrero-Navarro et al., 2022; Logan et al., 2021; Qiao et al., 2024). As such, age-related loss of neuronal LAL was reported to promote neuronal lipid accumulation and neurodegeneration in mouse and post-mortem human brains (Barnett et al., 2025; Moriwaki et al., 2024). Induced NPC1 loss resulted in the buildup of CE, cholesterol crystals, and gangliosides, causing lysosomal expansion and ameboid morphology in zebrafish microglia upon high neuronal apoptosis *in vivo* (Zareba et al., 2024). Functional consequences of NPC1 loss included increased myelin uptake by transgenic mouse microglia, but its decreased trafficking to lysosomes in an *ex vivo* assay (Colombo et al., 2021). Linked to aging brain and frontotemporal dementia, the loss of PGRN induced dysregulation in lysosomal maturation and LD accumulation in mice and human induced microglia (iMG) (Matsuwaki et al., 2011; Sung et al., 2024). An *in vitro* CRISPR-Cas9 assay validated the transcriptional and functional similarity between these LD-accumulating PGRN-KO mouse microglia and aged mouse LDAM (Marschallinger et al., 2020). Consequently, lysosomal acidity is also emerging as a target for modulation of autophagic clearance and innate immune signaling regulation in microglia (Moriwaki et al., 2024; Quick et al., 2023). Pharmacological re-acidification of lysosomes with ketotifen, an H1 antihistamine, mitigated memory impairment and restored a homeostatic microglial profile in aged mouse hippocampus and cerebral cortex (Barrella et al., 2026). Collectively, these findings underscore tight control of lysosomal pH as a fundamental component of lysosomal function that may represent a potential target for aged microglia/LDAM modulation.

An earlier study identified myelin debris as a primary contributor to LF/LD accumulation in microglia from the aged mouse white matter (Safaiyan et al., 2016). The structure of myelin sheaths, whose continuous turnover is co-regulated by microglial clearance of fragmented and excess myelin, is dominated by lipids (e.g., cholesterol, phospholipids, and glycolipids) (Montilla et al., 2025; Safaiyan et al., 2016). Aging increases the fragmentation and decreases the structural stability of myelin (Dimovasili et al., 2025; Safaiyan et al., 2016). This is supported by the observation of an altered myelin lipidome in the corpus callosum of aged rhesus monkeys, defined by decreased levels of ceramides and PUFA as well as increased lipid saturation (Dimovasili et al., 2025). Recapitulating aging effects, induced demyelination or genetic blockade of microglial lysosomal function in adult mice triggered an enlargement of late endosomes/lysosomes and LF/LD load (Marschallinger et al., 2020; Safaiyan et al., 2016, 2021). TREM2 mediates adaptation of microglial lysosomal function and lipid metabolism to lipid-rich environments (Nugent et al., 2020; Poliani et al., 2015; Safaiyan et al., 2021). Microglia-specific TREM2 KO in mice disabled DAM conversion and amplified buildup of CE-enriched LD in the context of cuprizone-induced demyelination (Nugent et al., 2020). Functionally, impaired cholesterol flux was tied to microglial deficiency in phagocytosis of myelin and support of remyelination in aged mice (Cantuti-Castelvetri et al., 2018). During physiological aging, isolated naïve TREM2+ microglia showed only mildly upregulated cholesterol metabolism compared to young adult murine microglia (Nugent et al., 2020). While this could reflect a lower cholesterol burden with aging (vs. a demyelination pathology), this outcome may also be contributed to by altered levels of phagocytic markers in a region- and/or microglia state-dependent manner. Thomas et al. (2022) reported that relative to adults, aged murine microglia downregulate receptors associated with phagocytosis, e.g., presenting ∼22% decrease in TREM2. Counterintuitively, pro-phagocytic white matter-associated microglia (WAM) were shown to emerge with aging in a TREM2-dependent manner, and display upregulation of cholesterol and lysosomal pathways (Safaiyan et al., 2021). Similarly, another paper reported aged murine and human microglial state displayed a proteomics signature highly enriched in TREM2 and senescence markers (e.g., p16, p19, p53), but distinct from the classical DAM signature (Rachmian et al., 2024). These data further align with the emergence of a microglial cluster enriched for *Trem2* in the cerebral cortex and striatum of aged female mice (Allen et al., 2023). In parallel, the aging-related hippocampal LDAM signature was found to differ from other previously reported aging-related TREM2-dependent DAM-like states (Marschallinger et al., 2020). This comparison involved the absence of differential regulation of representative gene, such as *Trem2* or *ApoE*, in aged LDAM (Marschallinger et al., 2020). The aging-related decrease in phagocytosis likely does not occur uniformly across the brain, and overall levels might be regulated by timeframe or contextual cues, e.g., myelin and cellular composition (Grabert et al., 2016; Thomas et al., 2022). Simultaneously, the content of aging LDAM LDs was found primarily to consist of glycerolipids (e.g., TAGs), while cholesterol was represented at only lower levels (Marschallinger et al., 2020). The bioenergetic profile of the young adult murine hippocampus and cerebellum was reported to be more enriched in pathways associated with energy production (glycolysis, electron transport chain, ATP synthesis and redox activity), metabolic regulators (PPARγ), and antioxidant responses compared to the striatum or cerebral cortex (Grabert et al., 2016). Authors reasoned this represents an increased functional responsiveness to immune stimuli in these regions. On contrary, aging was associated with a selective loss of immunoregulatory phenotype in the hippocampus versus an increase in the cerebellum (Grabert et al., 2016; Hart et al., 2012). Coinciding with LDAM-related dysfunction in phagocytosis, aged hippocampal microglia displayed gene reductions in pathways related to process mobility, cell body mobility, cytoskeletal organization, endocytosis, and immune functions (ligand recognition, processing and presentation) in the hippocampus (Grabert et al., 2016; Marschallinger et al., 2020). These findings indicate phagocytosis of large lipid-rich substrates may not represent the main pathway driving the aging LDAM profile.

During physiological aging, LDAM cells positive for a neutral lipid dye BODIPY were identified at over 50% occurrence in the hippocampus of 20-month-old mice, while rarely present in other brain regions (Marschallinger et al., 2020). In pathological inflammatory conditions associated with detrimental buildup of protein aggregates, such as AD or Parkinson’s disease (PD) (Han et al., 2025; Haney et al., 2024) and stroke-related hypoxia (Arbaizar-Rovirosa et al., 2023), LDAM-like prevalence further increased in abundance and across the brain regions. Moreover, the APOE isoform linked to AD risk (APOE ε4) most prominently exacerbated LD dysmetabolism in human iMG exposed to fibrillar Aβ peptide *ex vivo* compared to the other isoforms (Haney et al., 2024). Associated with neurotoxic responses (Haney et al., 2024), LDAM thus likely serve as both a consequence and driver of aging and neurodegenerative pathology in vulnerable individuals. In line, aging has been defined by chronic low-grade systemic inflammation (“inflammaging”) across tissues, which contributes to immune cell dysfunction (Franceschi et al., 2018; Mosher and Wyss-Coray, 2014; van Beek et al., 2019). Microglia/macrophages were proposed to adopt a “*primed*” phenotype, characterized by an increased baseline inflammatory cytokine production and exaggerated response to subsequent immune stimuli (Mosher and Wyss-Coray, 2014; Sierra et al., 2007). Coincidentally, LD buildup is also a reported feature of the acute immune response of myeloid cells to inflammation- or stress-related stimuli (e.g., bacteria), linked with activation of interferon (IFN) pathways and TLR signaling (Arbaizar-Rovirosa et al., 2023; Marschallinger et al., 2020; Monson et al., 2018; Pereira-Dutra et al., 2019). Considering this mechanistic overlap, LDAM have been hypothesized to represent a maladaptive adaptation to innate immune triggers and inflammaging (Haney et al., 2024; Marschallinger et al., 2020). Accordingly, the LPS pathway was identified as a primary driver of the differential gene expression between murine aged LD-enriched and LD-low microglia (Marschallinger et al., 2020).

### 3.2 The lipid axis of metabolic and functional reprogramming in aged LDAM

As reported in primary mouse and human induced pluripotent stem cells (iPSC)-derived microglia, immune stimulation triggers an energetic shift from OXPHOS to aerobic glycolysis (Cheng et al., 2014, 2021; Hu et al., 2020; Sabogal-Guaqueta et al., 2024). Instead of entering the TCA cycle, glucose-derived pyruvate is thus shuttled toward a high-rate production of ATP and substrates for lipid and nucleotide synthesis (Vander Heiden et al., 2009). This provides rapid energetic, metabolic and structural component resources for microglial adaptations associated with acute immune response. In conditions of hypoxia or TLR stimulation, macrophages upregulate glycolysis and interleukin 1-beta (IL-1β) release via the activation of hypoxia-inducible factor 1-alpha (HIF-1α) pathway (Blouin et al., 2004; Cheng et al., 2014; Tannahill et al., 2013). Under a transcriptional control of mTORC1, HIF-1α is a transcription factor for glucose transporter, glycolytic enzyme and inflammatory cytokine gene expression (Blouin et al., 2004; Cheng et al., 2014). In primary mouse microglial cells exposed to LPS, this glycolytic reprogramming exerts TCA cycle interruption and accumulation of its intermediate molecules, such as succinate or citrate (Nair et al., 2019). Succinate elevation stabilizes HIF-1α and prolongs its activation, which enables a sustained maintenance of glycolytic metabolism during immune response (Antignano et al., 2023; Ding et al., 2013; Minhas et al., 2021).

In resemblance to the ‘reactive’ microglia state, aged murine microglia were reported to display an increase in rate and capacity of glycolysis *in vitro* (Mela et al., 2020) – an effect postulated to be driven by chronic low-grade systemic inflammation. As opposed to the metabolic flexibility of young adult microglia in utilization of alternative energy sources, aged myeloid cells were found to become highly dependent on glucose (Bernier et al., 2020a; Minhas et al., 2021). Nonetheless, the relative dynamics of glycolysis and its role in the metabolism of aged microglia remains a subject of ongoing scientific discourse (Goyal et al., 2017). Given the extensive crosstalk between glucose and lipid pathways, the age-related changes in lipid flux likely play a vital role in regulation of glucose metabolism in aged microglia. Notably, an impaired glucose flux was triggered by an aging-associated rise in the levels of pro-inflammatory eicosanoid PGE2 in cultured human and mouse aged myeloid cells (Minhas et al., 2021). By acting on PGE2 receptor E2 (EP2), elevated PGE2 was shown to aid glucose sequestration into storage glycogen (Minhas et al., 2021). Disrupted glucose delivery initiated/exacerbated the buildup of TCA cycle intermediates and decreased the mitochondrial respiration, which sequentially resulted in an energetic deficiency and maladaptive signaling (Minhas et al., 2021). Pharmacological EP2 inhibition reduced circulating pro-inflammatory markers and percentage of hippocampal CD68+ microglia, while restoring memory performance in aged mice to levels observed in young adults (Minhas et al., 2021). The conversion of arachidonic acid into PGE2 is enzymatically facilitated by cyclooxygenase-2 (COX-2) (Claycombe et al., 2002; Wu et al., 1998). While the cause of the aging-related PGE2 increase is unclear, elevation of intracellular ceramide with aging was associated with the transcriptional upregulation of COX-2 (Claycombe et al., 2002; Wu et al., 1998). Ceramides can be generated through the sphingomyelinase-catalyzed transformation of membrane sphingomyelins, lysosomal hydrolysis of complex phospholipids (the salvage pathway), or *de novo* synthesis from palmitoyl-CoA and serine in ER stimulated by elevated free FA (McInnis et al., 2024; Pujol-Lereis, 2019). It is thus plausible aged microglia accumulate ceramide as a consequence of their dynamic activity requiring membrane reorganization/repair, metabolic adaptations, and phagocytosis of membrane debris throughout their life. This buildup may be enhanced by an impairment of enzymes involved in ceramide metabolism such as acid sphingomyelinase, which is stimulated by oxidative stress or tumor necrosis alpha (TNF-α) (Crivelli et al., 2023; Cutler et al., 2004; Li et al., 2012; McInnis et al., 2024).

At present, mechanistic evidence for the involvement of the PGE2-EP2-HIF-1α signaling in LDAM remains lacking. Conversely, the transcriptomes of LD^high^ microglia from the aged and PGRN-KO mouse models exhibited an overlap in a differential expression of genes involved in prostaglandin synthesis pathway (Marschallinger et al., 2020). There was a downregulation in PGE2 receptor 4 (EP4) gene expression, an effect that was previously shown to accompany increased PGE2-EP2 signaling and lipid peroxidation in LPS-exposed cultured mouse microglial cells (Bonfill-Teixidor et al., 2017; Niraula et al., 2023; Shi et al., 2010). Additionally, RNA-seq of LD^high^ vs LD^low^ aged LDAM revealed an enrichment in the gene expression for ATP citrate lyase (ACLY) (Marschallinger et al., 2020). Connecting carbohydrate and lipid pathways, ACLY promotes conversion of the TCA-derived citrate into cytosolic Acetyl-CoA, which provides building blocks for *de novo* FA, cholesterol and LD synthesis (Covarrubias et al., 2016). Furthermore, in *post-mortem* cortical tissue of human patients with AD, LDAM-like cells were associated with upregulation of ACSL1, which converts free FA into Acyl-CoA and TAGs (Haney et al., 2024; Victor et al., 2022). In response to LPS, ACSL1^high^ microglia isolated from a mouse model of a Parkinson’s disease pathology showed subcellular expression of ACSL1 localized primarily to the ER, a prime spot for LD formation (Han et al., 2025). Moreover, microglial ACSL1 expression forms a positive feedback loop with NF-κB signaling (Han et al., 2025), predicting LD buildup as a conclusive outcome of a spectrum of innate immune activators (Claes et al., 2021; Haney et al., 2024). Intriguingly, the aged LDAM profile also displayed gene expression upregulation in FAO pathway (Marschallinger et al., 2020). FAO is dependent on oxidized nicotinamide adenine dinucleotide (NAD+), with LDAM displaying a higher oxidized-to-reduced form of NAD ratio (Marschallinger et al., 2020). This seemingly contrasts with low levels of *de novo* NAD+ synthesis previously reported in *in vitro* murine and monocyte-derived human macrophages in the context of aging (Minhas et al., 2019). These findings suggest aged LDAM might be utilizing the citrate shunt and LD formation to recycle the energetic fuel for FAO in order to compensate the mitochondrial ATP production and to maintain its membrane potential. Furthermore, the TCA intermediate buildup might represent an outcome of aged mitochondrial dysfunction and decreased capacity to utilize non-glucose substrates. Moreover, a prolonged duration of this metabolic strategy is likely detrimental to the cell, as LD membranes can also serve as sites for PGE2 synthesis via localized COX-2 activity (de Almeida et al., 2018; Melo et al., 2011; Stephens and Johnson, 2025). Their buildup in LDAM suggests a propensity for a further exacerbation of eicosanoid synthesis, glucose/TCA cycle dysfunction, and maladaptive programming (Antignano et al., 2023).

The lysosomes seemingly represent a crucial rate-limiting step in this LD metabolism. In line, the ultrastructure of aged LDAM demonstrates lysosomal location of LD bodies, indicating lipophagy as a major pathway for the LD turnover (Burns et al., 2020; Marschallinger et al., 2020). This is supported by a concomitant upregulation of endolysosomal and autophagic pathways observed in LDAM- or LF-accumulating aged murine microglia (Burns et al., 2020; Marschallinger et al., 2020). While lipophagy is thus enhanced, aging and accumulation of undigestible debris affects the efficiency of lysosomal digestion in aged LDAM. The full extent of the impact of these alterations on the rate and capacity of lipid hydrolases or the ratio of lysosome-generated/released lipid species, however, needs to be further determined. In parallel, mTORC1, which fine-tunes microglial autophagic capacity and lysosomal activity, was shown to be upregulated in primed microglial cells isolated from aged mice and humans (Keane et al., 2021). In LDAM, the mTORC1 dysregulation could stem from an impaired sensing of the cell’s nutritional status (due to excess of FA) and/or elevated innate immune stimulation. Nevertheless, this angle remains yet largely underexplored.

Aged murine LF-enriched microglia as well as hippocampal LDAM signature were characterized by a significant upregulation of genes associated with the production of nitric oxide and reactive oxidative species (ROS) (Burns et al., 2020; Marschallinger et al., 2020). As FAO is an oxygen-dependent process, its elevation can contribute to ROS production (Schönfeld and Reiser, 2013). Oxidative stress is most notably connected to cell stress responses mediated by NF-κB, which promotes release of pro-inflammatory cytokines, including TNF-α, IL-1β, or IL-6 (Ishihara and Itoh, 2023). Mitochondria-derived ROS can also serve as a stabilizing factor of HIF-1α (Chandel et al., 2000). Conversely, rapamycin-induced mTORC1 inhibition upregulated NF-κB-mediated transcription in human monocytes and mouse macrophages (Linke et al., 2017; Weichhart et al., 2008). On the contrary, rapamycin was linked to anti-inflammatory outcomes and beneficial impact on murine lifespan and cognitive performance in neurodegenerative conditions (Harrison et al., 2009; Spilman et al., 2010). As such, genetic mTORC1 ablation in aged mice replicated NF-kB upregulation and priming; however, it also showed a decrease in 4EBP1-eIF4E-mediated translation of cytokine mediators (Bermudez et al., 2023; Keane et al., 2021). Similarly, inhibition of PI3K/AKT, an upstream regulator of mTORC1, reduced lipid-lysosome dysregulation and inflammatory signaling in APOE4/4 iMG exposed to fAβ *ex vivo* (Haney et al., 2024). mTORC1 thus represents an attractive LDAM modulation target, albeit studies also indicate that intervention outcomes may be influenced by the delivery modality, duration and context (Keane et al., 2021). Alternatively, LD accumulation in primary microglial cells after LPL KO was associated with reduced PPARγ expression (Loving et al., 2021). Experimental PPARγ activation was linked to an inhibition of microglial pro-inflammatory profile after traumatic brain injury in mice (Pearson et al., 2024). In a similar fashion, LXR signaling was reduced due to toxic overload of cholesterol and impaired synthesis of its oxidized sterol ligands in aged mice after a severe demyelination injury (Cantuti-Castelvetri et al., 2018). Treatment with an LXR agonist upregulated cholesterol efflux-associated gene expression, which facilitated a decrease in cholesterol/cholesterol crystal buildup in myeloid cells and promoted lesion recovery (Cantuti-Castelvetri et al., 2018).

Another pathway contributing to pro-inflammatory activity of aged LDAM may be mediated via the NLRP3 inflammasome and its caspase-1-mediated IL-1β release (Cantuti-Castelvetri et al., 2018). In a crosstalk with other cell stress response pathways, TLR and/or NF-κB activation can serve as priming factors for NLRP3 assembly (Iacano et al., 2019; Li et al., 2021). In an aged mouse model of demyelination, damage to lysosomal membranes triggered leakage of its contents and activation of NLRP3 (Cantuti-Castelvetri et al., 2018). So far, this event was mainly linked to the formation of rigid, insoluble and poorly degradable cholesterol crystals in lysosomes of aged microglia exposed to myelin overload (Cantuti-Castelvetri et al., 2018; Gabandé-Rodríguez et al., 2020). While aging hippocampal LDAM display low esterified cholesterol in LDs and absence of cholesterol crystals (Marschallinger et al., 2020), a combination of oxidative stress and lysosomal dysfunction due to lipofuscin accumulation may progressively create an environment favoring NLRP3 activation.

Similarly, excess free FA in combination with oxidative stress increases the risk of lipid peroxidation (Loo et al., 2025; Mortensen et al., 2023). Subsequent alteration in the fluidity and dynamics of the cytoplasmic membrane may impact microglial effector functions such as transmembrane molecule transport, signaling, ligand recognition, or phagocytosis (Rojo et al., 2014). Found to be increasingly elevated in the brains of mice and humans, excess labile iron is a potent inducer of ROS production and lipid peroxidation (Sato et al., 2022; Winterbourn, 1995). In aging-associated human white matter injury, build-up of PLIN2+ LD in microglia was accompanied by enrichment in light chain ferritin, an iron storage protein, and an increased susceptibility to ferroptosis (Adeniyi et al., 2023). Investigation of the role of iron-mediated ROS production in LD turnover of aged microglia is currently under review.

Within the broader cellular network, alterations of the lysosomal-lipid-mitochondria-LD axis are not an isolated cellular element, with oxidative stress and altered lipid flux likely simultaneously impacting other cell components. Both phagocytic and non-phagocytic aged murine microglia presented an elevated expression of genes associated with cell cycling and DNA repair, indicating nuclear stress and increased metabolism (Thomas et al., 2022). Isolated aged mouse microglia, particularly highly enriched in lipofuscin and lysosomal storage, also showed an increased positivity for Annexin V, an apoptotic cell death marker (Burns et al., 2020). Given its central role in lipid metabolism, the ER stress response was proposed as a contributing mechanism associated with lipid accumulation in pro-inflammatory foamy macrophages during atherosclerosis (Sukhorukov et al., 2020). In an AD pathology mouse model, microglia-specific autophagy disruption reduced the expression of ER stress response genes and triggered oxidative stress (Cai et al., 2025). Resulting lipoperoxidation and ferroptosis decreased microglial capacity to handle Aβ plaques *in vitro* (Cai et al., 2025). While ER stress response activation and oxidative stress did not alter microglial density in normally aged transgenic mice, their presence indicates an increased vulnerability of aged microglia to innate immune stress factors (Cai et al., 2025).

### 3.3 Crosstalk of lipid-lysosome axis and epigenetic remodeling

Epigenetic mechanisms (e.g., DNA or histone modification) mediate acute immune responses and lay the grounds for the formation of microglial immune memory and priming across the lifespan (Li et al., 2023; Nirakis et al., 2025; Tiwari et al., 2024). Focus has been on a family of histone acetylases (HDAT) and deacetylases (HDAC), which modulate chromatin compaction and activity of cytosolic enzymes through removal of acetyl groups from lysine residues (Datta et al., 2018; Ding, 2025). Emerging evidence indicates a therapeutic potential for the modification of lipid-associated gene expression via regulation of histone acetylation status. For instance, in a murine model of spinal cord injury, acetylation of microglial histone H3 at lysine 27 (H3K27ac) was reported to modulate transcription at cholesterol metabolism-related gene loci (Huang et al., 2023; Qiao et al., 2026). White matter microglia in 15-month-old mice decreased chromatin accessibility at immune- and lipid metabolism-associated gene promoters after a myelin injury compared to young mice (Cantuti-Castelvetri et al., 2018; Tiwari et al., 2024). In that regard, ablation of HDAC1 and -2 improved microglia-mediated lipid clearance and lesion recovery in aged mice (Tiwari et al., 2024). Rather than priming, these epigenetic alterations were hypothesized to promote gradual aging-induced loss of microglial ability to initiate a coordinated damage-specific immune response (Tiwari et al., 2024). Compared to disease contexts, there is yet limited understanding of how epigenetic alterations contribute to physiological aging states in microglia. Nonetheless, increased expression of HDAC1 and -3 was positively correlated with markers of microglial activity elevated in aging (CD68, IBA1, ITGAM) and senescence (p21, p16) in a mouse model of induced senescence as well as aged human hippocampus (Auzmendi-Iriarte et al., 2022). In cytosol, hyperacetylation of fatty acid synthase after traumatic brain injury was linked to LD buildup and pro-phagocytic activity in primary adult microglia *ex vivo* (Zhang et al., 2025). HDA3 was identified as key posttranslational regulator of this *de novo* lipogenetic pathway under physiological conditions (Zhang et al., 2025). These findings indicate variable epigenetic programming of the lipid metabolism underlying aging LDAM and aging pro-phagocytic microglial states. In relation to aging LDAM, ACLY-generated Acetyl-CoA can also serve as a substrate for histone acetylation (Lauterbach et al., 2019). A similar pathway was also described as part of LPS-induced TLR activation and immune response in murine BMDMs *in vitro* (Lauterbach et al., 2019). From this perspective, epigenetic programming and transcriptional dynamics form an interactive network with metabolic processes in fine-tuning of microglial effector functions. Conceptually, cumulative adaptations of this metabo-epigenetic axis in response to innate or environmental triggers across the long life of microglia (Benayoun et al., 2019; Costa et al., 2021; Li et al., 2023; Nirakis et al., 2025) may be driving their sustained priming and loss of functional plasticity with aging.

To sum up, the aging LDAM state is hypothesized to present a dysfunctional microglial adaptation to regional immune and inflammatory challenges. With the hippocampus presenting a high degree of experience-driven synaptic plasticity, the immunosensitive hippocampal microglia may be at an increased risk of damage accumulation (Keren-Shaul et al., 2017). Prolonged demand for microglia-mediated housekeeping and debris clearance due to widespread and parallel changes across tissue may progressively burden their lysosomal and energetic functions. Evidence points to LDAM displaying a pronounced lipogenic-lipolytic metabolic loop that may be hypothetically attempting to restore the balance between the declining lysosomal clearance capacity, repair and respiration, and increasing functional demands of the aging parenchyma. This maladaptive survival-like strategy likely contributes to altered engagement of aging LDAM with surrounding cells and involvement in processes such as synaptic plasticity.

## 4 Neuron-microglia lipid crosstalk and synaptic plasticity in aging

It is well-established that in the adult brain, microglia dynamically monitor synapses and perform their low-scale refinement in an activity-dependent manner at both pre- and post-synaptic sites (Nguyen et al., 2020; Paolicelli et al., 2011; Schafer et al., 2012; Tremblay et al., 2010; Wake et al., 2009). This interaction occurs via release of trophic factors and cytokines, remodeling of synapse-stabilizing perineuronal nets or phagocytosis of cell debris/synapses. The gradual weakening and loss of synaptic connections is a major contributing factor to aging-related cognitive dysfunction (Bezprozvanny and Hiesinger, 2013; Cribbs et al., 2012; Tremblay et al., 2012). Aged microglia, LDAM particularly, were reported to display reduced motility and decreased phagocytic capacity for myelin fragments, zymosan, *Escherichia coli* particles, or Aβ *in vitro* and *in vivo* (Adeniyi et al., 2023; Arbaizar-Rovirosa et al., 2023; Gabandé-Rodríguez et al., 2020; Haney et al., 2024; Hefendehl et al., 2014; Marschallinger et al., 2020; Zhao et al., 2025). However, the impact of this metabolic programming on its interactions with synaptic elements is not yet fully understood, raising several distinct possibilities.

Microglia secrete complement component 1 (C1q) as a tool for phagocytic removal of opsonized debris, pathogens and weak synapses (Shi et al., 2015; Stephan et al., 2013). Recognition of C1q-tagged synapses via the complement component 3 (C3)-C3 receptor (CR3) pathway initiates microglial phagocytosis of synaptic debris (Schafer et al., 2012; Stevens et al., 2007). Protein levels of C1q were shown to significantly increase in the brain of aged mice and humans, particularly in the hippocampus and in proximity of synapses (Stephan et al., 2013). Abnormal C1q deposits were hypothesized to represent a buildup of damaged synapses with increased susceptibility to microglia-mediated clearance (Chung et al., 2016; Mukhtar et al., 2025). Additionally, C1q KO prevented the decline in mouse hippocampus-related behaviors (Stephan et al., 2013). Previously, proteomic analysis showed that microglia isolated from aged mice accumulate synapse-related proteins with slower age-dependent degradation kinetics at a higher degree than young mouse microglia (Guldner et al., 2026). The internalization of neuronal exosomes and selective targeting of damaged synapses were proposed as underlying mechanisms of this outcome that progressively contribute to detrimental microglial programming (Guldner et al., 2026). Accordingly, the ultrastructural profile of microglia from sensory cortices of aged mice presented an age-dependent increase in synaptic element-like phagocytic inclusions alongside LF and lipid bodies (Tremblay et al., 2012). Alternatively, this effect could represent an outcome of reported deficiency in phagocytic capacity of aging LDAM (Marschallinger et al., 2020). Given the reported decrease in brain levels of C3 protein with aging, C1q presence was additionally suggested to impact circuitry in a non-classical complement manner (Stephan et al., 2013). Its aging-associated increase was linked to a repressive microglia-mediated regulation of neuronal protein translation in adult mouse brain tissue (Scott-Hewitt et al., 2024). The brain-wide proteomic analysis revealed alterations in neuronal pathways associated with cytoskeletal organization and mitochondrial function, which are essential for synaptic function (Scott-Hewitt et al., 2024).

In reflection of distinct brain region-dependent impact of aging (Grabert et al., 2016; Morrison and Baxter, 2012), a modest elevation of C3 protein levels was reported in hippocampal homogenates of middle-aged male mice (Shi et al., 2015). Upregulation of complement cascade-associated proteins, such as C1q, C3, or C3R, was further shown in the hippocampal tissue of aged humans (Cribbs et al., 2012). In mice, these effects coincided with a synaptic loss and decline in synaptic function in hippocampal CA3 region (Shi et al., 2015). C3-deficient mice, however, were protected from the age-dependent loss of CA3 synapses (Shi et al., 2015). More recent experiments have highlighted the role of C3/C3R pathway in the regulation of microglial lipid metabolism. In a mouse model of Aβ pathology induced by human Amyloid precursor protein (APP)^NL–G–F^ mutation knock-in (KI), the plaque-associated microglia were marked by enrichment in C3aR expression, HIF-1α signaling, lipid metabolism and LD accumulation (Gedam et al., 2023). Similarly, ACSL1+ LDAM-like microglia were frequently observed in Aβ plaque proximity in post-mortem brain tissue of patients with AD (Haney et al., 2024). Compared to the wild-type control, C3aR ablation attenuated HIF-1α staining and reduced accumulation of BODIPY^+^ LD in response to hypoxia or LPS in a primary culture of neonatal murine microglia (Gedam et al., 2023). Ultimately, this was also associated with an elevated trafficking/recycling of microglial phagocytosis-associated receptors (TREM2, CD36) and enhanced phagocytosis of Aβ fibrils under hypoxia *in vitro* (Gedam et al., 2023). Furthermore, the volume of microglia-engulfed postsynaptic density protein 95, a pre-synaptic marker, was significantly lower after C3aR ablation in APP-KI mice (Gedam et al., 2023). A mechanistical link between aging LDAM and C3aR-enriched microglia has not been explored yet; however, the overlap between their reported metabolic and functional features warrants further investigation. Moreover, this phagocytic profile displays a certain substrate specificity, which may be driven by lysosomal dysfunction in terms of phagocytic receptor recycling or maintenance of cytoskeleton flexibility. Genetic impairment of a cytoskeleton scaffold protein shifts mouse microglia toward a morphology favoring thin F-actin cytoplasmic filopodia and limited motility *in vitro* (Kessels et al., 2025). Investigated in macrophages of Drosophila embryos *in vivo*, this filopodial phagocytosis was proposed to support clearance of spatially constrained and smaller targets (Davidson and Wood, 2020), such as are putatively synaptic elements (Kessels et al., 2025). On an intracellular level, an age-related increase in F-actin cytoskeleton assembly impaired lysosomal vesicle trafficking and autophagy in the aged fly brain *in situ* (Schmid et al., 2024). Transcriptomic analysis of acutely isolated aged human cortical microglia revealed alterations in actin cytoskeleton-related genes, including profilin 1 (Galatro et al., 2017). Akin to LDAM, conditional loss of microglial profilin 1 in adult murine microglia reduced their motility *in vivo* and triggered a pro-inflammatory microglial profile positive for CD68 (Portugal et al., 2025). These alterations were accompanied by an inhibitory synapse loss in the mouse prefrontal cortex (Portugal et al., 2025). A dynamic cytoskeleton required for phagocytic processes is indicated as an essential regulator of LD metabolism (Davis et al., 2023; Liang et al., 2025). Age-related decline in cytoskeletal organization pathways reported in aged hippocampal microglia (Grabert et al., 2016) alongside changes in lipid composition of organizing lipid rafts may be linked to phagocytic dysfunction and LD accumulation in aged LDAM. More research is, however, needed to determine any directionality.

Beyond direct contacts, pro-inflammatory signaling of aged LDAM may also negatively modulate synapses by sustaining neuroinflammation (Burns et al., 2020; Marschallinger et al., 2020; Patterson, 2015). While baseline cytokines, such as IFN-ɣ, TNF-α, and IL-1, are required for dynamic adaptations of synaptic structure, transmission and plasticity, the prolonged and elevated levels may induce neurotoxicity and synaptic loss (Sierra et al., 2007; Zipp et al., 2023). Hypothesized to serve an initial tissue preservatory role by entering a cell cycle arrest, senescent-like microglia are thought to drive CNS aging and vulnerability to aging-related pathologies through a NF-κB-regulated pro-inflammatory secretory profile (SASP) (Carr et al., 2025; Coppé et al., 2008; Hu et al., 2017). There are also indices that exocytosis of lysosomal contents, notably by microglia/macrophages, may relate to the propagation of potentially neurotoxic substrates (Rovira et al., 2022). Conditioned media from LPS-stimulated human iPSC-derived microglia enhanced neuronal network activity *in vitro* by elevating neuronal LD turnover in an APOE isoform-dependent manner (Verduzco Espinoza et al., 2025). Conditioned media from LD^high^ APOE4/4 or APOE3/3 human iMG induced tau phosphorylation and caspase activation in human iPSC-derived neurons; an effect not present if APOE was knocked out (Haney et al., 2024). In the same experiment, LD^high^ iMG media induced neuronal accumulation of TAG, indicating alteration in lipid flux (Haney et al., 2024). A recent analysis of APOE-lipoproteins produced by primary adult mouse microglial cells expressing the human *ApoE* gene reported enrichment in CE compared to astrocytic lipoproteins (Strickland et al., 2026). Their proteomic signature was primarily enriched in proteins associated with lipid metabolism and the complement pathway, with the latter particularly increased with the APOE ε4 isoform (Strickland et al., 2026). Under physiological conditions, microglia are not significant producers of APOE in the CNS compared to astrocytes (Ulrich et al., 2018) and the murine LDAM signature was similarly not associated with ApoE regulation (Marschallinger et al., 2020). However, disruption of LD metabolism in age-associated LDAM could hypothetically induce alterations in lipid and protein composition of secreted APOE-lipoproteins, which subsequently alter synaptic function (Wang N. et al., 2024).

In neurons, functional synapse maintenance relies on recycling of synaptic membranes and vesicles through the endo-lysosomal system and autophagy at both pre- and postsynaptic levels (Bezprozvanny and Hiesinger, 2013; Burrinha et al., 2023; Cuervo and Macian, 2014). Similar to microglia, the abundance of enlarged LAMP1-positive late endosomes increased within neurites and synaptic terminal compartments of aged mouse neurons *in vitro* and *in vivo* (Burrinha et al., 2023). Aging-related LF deposits were also more abundant in microglia and neurons compared to other cells, such as astrocytes and oligodendrocytes, in the rodent CNS (Dougnon and Matsui, 2025). Recent evidence indicates that neuronal dependence on metabolism of FA and LD may be greater than previously recognized, particularly in the synaptic compartment and in processes underlying neurotransmission and memory formation (Kumar et al., 2025; Saber et al., 2025; Zhang et al., 2026). Synaptic proteins were found to be progressively enriched in autophagic vesicles of the adult and aged mouse forebrain, indicating decline in lysosome-autophagy function (Kallergi et al., 2023). Given the neuronal sensitivity to oxidative stress generated from FA breakdown, lipid flux impairments may contribute to neuronal lipotoxicity (Saber et al., 2025; Verduzco Espinoza et al., 2025; Zhang et al., 2026). Accordingly, neuron-specific blockade of a TAG lipase impaired FA flux, which interfered with cognitive functioning and memory acquisition in mice (Akefe et al., 2024; Kumar et al., 2025; Saber et al., 2025). Neuronal lipid metabolism, which is upregulated during heightened network activity, depends on glial cells for supply, export and recycling of essential lipid species (e.g., cholesterol, lipoproteins) (Ioannou et al., 2019; Verduzco Espinoza et al., 2025; Wang N. et al., 2024). At the same time, these neuron-derived lipids drive functional and metabolic reprogramming of glia (Li et al., 2024a; Raj et al., 2017; Xu et al., 2025). This is indicated by neuronal transfer of unsaturated lipids triggering an LDAM-like phenotype in a mouse model of tauopathy (Li et al., 2024a). While representing a putatively protective mechanism for the removal of excess or damaged substrates from neurons, it could be contributing to microglial lysosomal burden and dysfunctional cell-cell interaction.

While the link between LD metabolism and microglial activity is evident, the external impact of its dysregulation (leading to LDAM formation) and the manner of its engagement with synapses leave lingering questions. Overall findings indicate this relationship is characterized by altered intercellular signaling, ligand recognition, metabolic control, and cytoplasmic membrane dynamics (Figure 4). Lipids and lipid-derived molecules, which exist in a constant and highly coordinated flux both within and between glia and neurons, are emerging as key drivers of these alterations. It is thus hypothesized that even subtle disturbances in lipid dynamic, when cumulative, may contribute to synapse decline, tissue aging, and late-onset cognitive dysfunction (Bezprozvanny and Hiesinger, 2013; Ioannou et al., 2019; Li et al., 2024a; Zhang et al., 2024).

**FIGURE 4:**
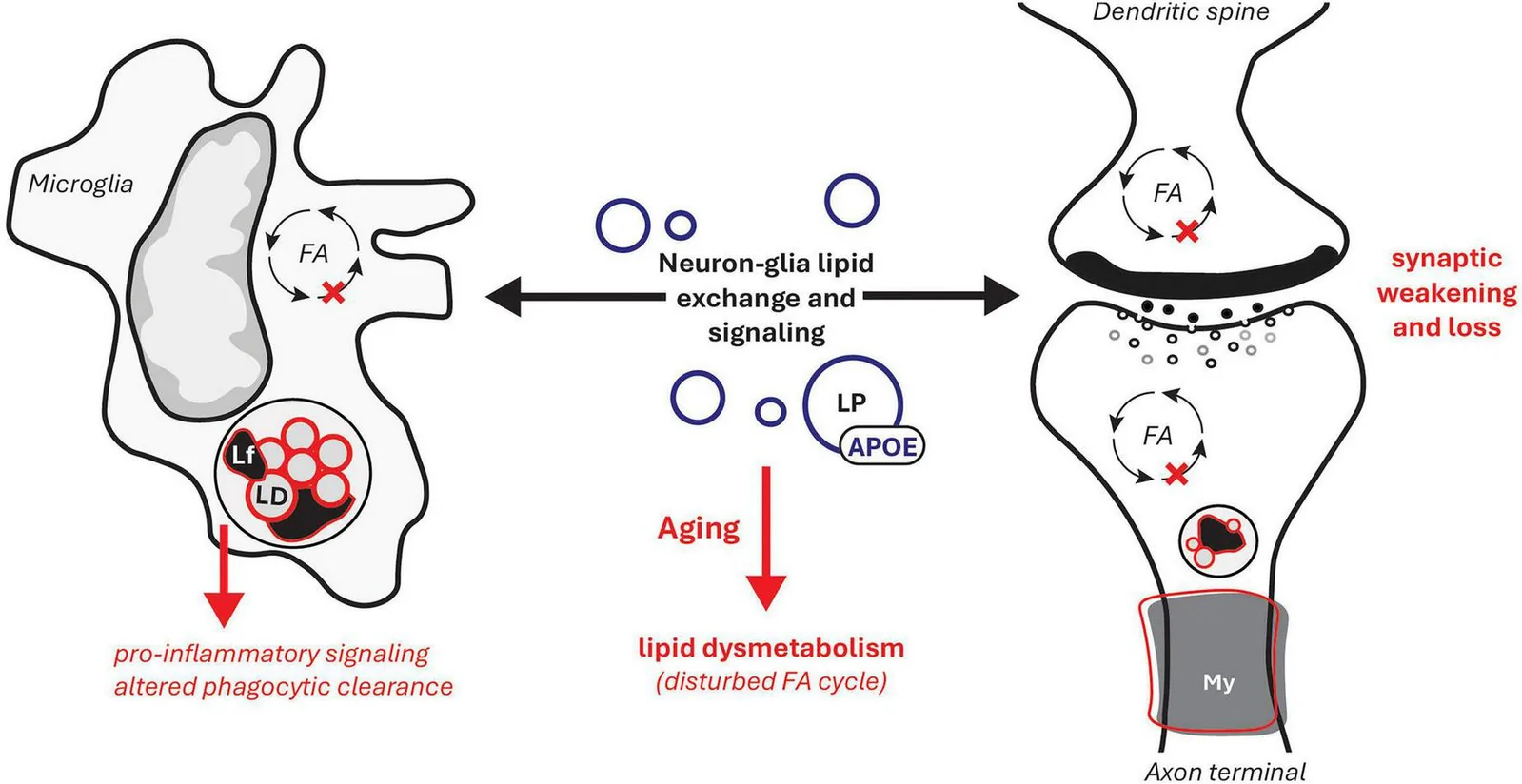
Illustrative representation of alterations in LDAM-neuron crosstalk in aging. The alterations in cellular lipid crosstalk and, adaptively, in intracellular lipid metabolism of neurons and glia accumulate across the lifespan. LD dysregulation in LDAM promotes its detrimental impact on synaptic integrity and function via pro-inflammatory signaling, altered lipid-mediated communication, and dysregulated phagocytosis-mediated myelin turnover and cell debris clearance. In a feedback loop, this cumulative metabolic reprogramming decreases microglial capacity for cellular stress handling and conditional adaptability. As a consequence of an impaired lipid flux and inflammation, synaptic strength weakens and may lead to synaptic loss. Cell structures: LD, lipid droplet; Lf, lipofuscin; My, myelin sheath. Molecules: APOE, apolipoprotein E; FA, fatty acid; LP, lipoprotein.

## 5 Conclusion

Microglial metabolic flexibility underlying their rapid, dynamic, and context-dependent functional shifts is highly dependent on lipids for ATP production, structural components, signaling and biosynthesis (Choi and Mook-Jung, 2022; Guerrero-Navarro et al., 2022; Traetta et al., 2025). Lysosomes are one of the crucial regulatory hubs for microglial lipid metabolism. Alterations in lipid flux and lysosomal dysfunction form a self-reinforcing loop that progressively leads to pro-inflammatory LDAM generation and sustained microglial fatigue. Tissue-level immune dysregulation and accumulation of maladaptive lipid-accumulating cells exacerbate aging-related neuroinflammation, loss of synaptic plasticity and neurodegeneration (Burrinha et al., 2023; Tremblay et al., 2012; Yang et al., 2022; Zhang et al., 2026).

The aging-related alterations of the microglial lipid-lysosomal network are likely a result of cumulative changes at the levels of epigenetics, metabolism, transcription and translation in the context of individual factors and genetic predispositions. The course of physiological brain aging displays a gradual advance and with unique spatiotemporal dynamics of its processes. However, most of our mechanistic insights into this process are derived from induced models of accelerated aging-like features, aging neurodegenerative disorders or originating in peripheral myeloid cells and tissues. Additionally, rodents, which are frequently utilized in these studies, rarely naturally develop human-like aging-related neurodegenerative conditions. This indicates species-specific differences in glial lipid handling and lysosomal capacities over the lifespan (Antignano et al., 2023; Carr et al., 2025; Galatro et al., 2017). Disentangling aging mechanisms and further incorporating other important biological factors, such as sex (Hahn et al., 2023; Khawaja et al., 2025; Zhang et al., 2023), will be critical to our further understanding of microglial aging.

The experimental restoration of microglial lysosomal function and reversal of LD accumulation has been showing beneficial outcomes on microglial phagocytic capacity and immune response (Arbaizar-Rovirosa et al., 2023; Barrella et al., 2026; Cantuti-Castelvetri et al., 2018; Choi et al., 2023; Haney et al., 2024; O’Neil et al., 2018). These were further associated with improvements in memory, lifespan and course of aging and aging-related pathologies in animal models, indicating putative targets for therapeutical intervention (He et al., 2024; Sung et al., 2025; Yang et al., 2014). Cautious approach is, however, necessary, as the dosage and timing of metabolism-targeting interventions seem to drive cell- and context-specific outcomes, which underscores the need for individual-based optimization in their application. Beyond pharmacological targeting of glial lipid-lysosomal axis, there is an increased recognition for the impact of systemic factors such as sleep, diet or exercise on these mechanisms. This highlights modifiable pathways through which lifestyle-based therapeutical strategies may support microglial resilience, synaptic function and myelin turnover (Albouery et al., 2020; Chauquet et al., 2024; González Ibáñez et al., 2023; Haynes et al., 2024; Zhuang et al., 2022).
